# Vagus Nerve Stimulator (VNS)-induced Severe Obstructive Sleep Apnea Which Resolved After the VNS Was Turned Off

**DOI:** 10.7759/cureus.6901

**Published:** 2020-02-06

**Authors:** Puja Gurung, Yash Nene, Manjamalai Sivaraman

**Affiliations:** 1 Sleep Medicine, University of Missouri, Columbia, USA; 2 Neurology, University of Missouri, Columbia, USA; 3 Sleep Medicine/Neurology, University of Missouri, Columbia, USA

**Keywords:** vagus nerve stimulator, obstructive sleep apnea, drug-resistant epilepsy

## Abstract

We present a young 21-year-old male with drug-resistant epilepsy who developed vagus nerve stimulator (VNS)-induced severe obstructive sleep apnea (OSA). Severe OSA resolved after the VNS was turned off.

## Introduction

The idea of vagus nerve stimulation (VNS) was conceived over 30 years ago when Zabara found that intermittent stimulation of the vagus nerve inhibits neural processes and can terminate seizures in dogs [1]. It has been in use for more than 30 years now, and has been proven to reduce the frequency and intensity of refractory seizures by 50% and also produces improvement in non-psychotic major depression [2]. There are only a few studies that have been conducted to study the effect of VNS on sleep-related breathing. In this article, we present a young male patient who had VNS-induced severe obstructive sleep apnea (OSA). This case report demonstrates the importance of being aware of this possibility in patients being considered for VNS implantation and potentially screening for OSA in these patients using polysomnography (PSG).

## Case presentation

A 21-year-old male presented to clinic with complaints of loud waxing-waning snoring, gagging in his sleep, multiple night time awakenings, and excessive daytime sleepiness with an Epworth Sleepiness Scale of 11. His past medical history was significant for drug-resistant epilepsy status after VNS implantation. But his seizures were uncontrolled despite being on high-setting VNS and multiple anticonvulsants. His medications list included clobazam 20 mg in the morning and 30 mg in the evening, lacosamide 250 mg twice daily, and zonisamide 300 mg in the morning and 200 mg at bedtime. His VNS was set at signal ON time of 30 seconds and signal OFF time of 1.1 minutes.

**Figure 1:**
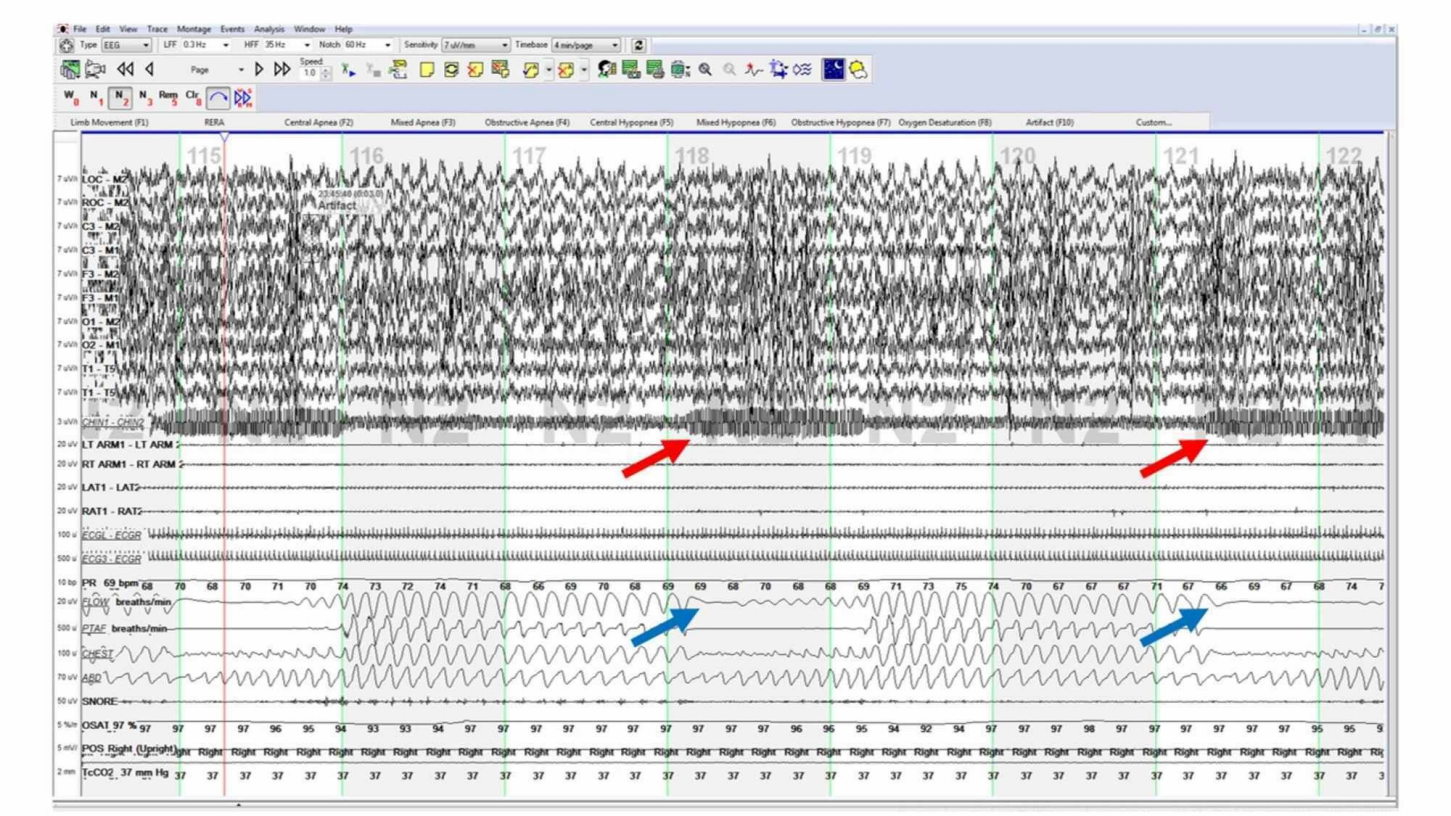
: Polysomnogram with the VNS turned ON showing increased chin EMG (red arrows) associated with mixed apneas (blue arrows). EMG, electromyography; VNS, vagus nerve stimulation.

He underwent a split night PSG (Figure 1). He had mild-to-moderate snoring and lowest oxygen saturation was 88%. Obstructive and mixed apneas were seen in all sleep stages and all positions during sleep. His apnea-hypopnea index (AHI) was 38.1/hour of sleep. He was started on continuous positive airway pressure (CPAP) and then bilevel positive airway pressure (BiPAP) the same night. Apneas persisted even with CPAP and BiPAP therapies. Interestingly, the obstructive and mixed apneic episodes were noted to be about 30 seconds in duration at fixed intervals of around 1one minute. These episodes correlated with recurrent increased chin electromyography (EMG) of same duration and at the same interval. Since his VNS setting ON time was 30 seconds and OFF time was 1.1 minutes, intermittent activation of the VNS during sleep seemed to trigger these episodes of apnea. No apneic events or other sleep disordered breathing events occurred independent of VNS stimulation. 

**Figure 2:**
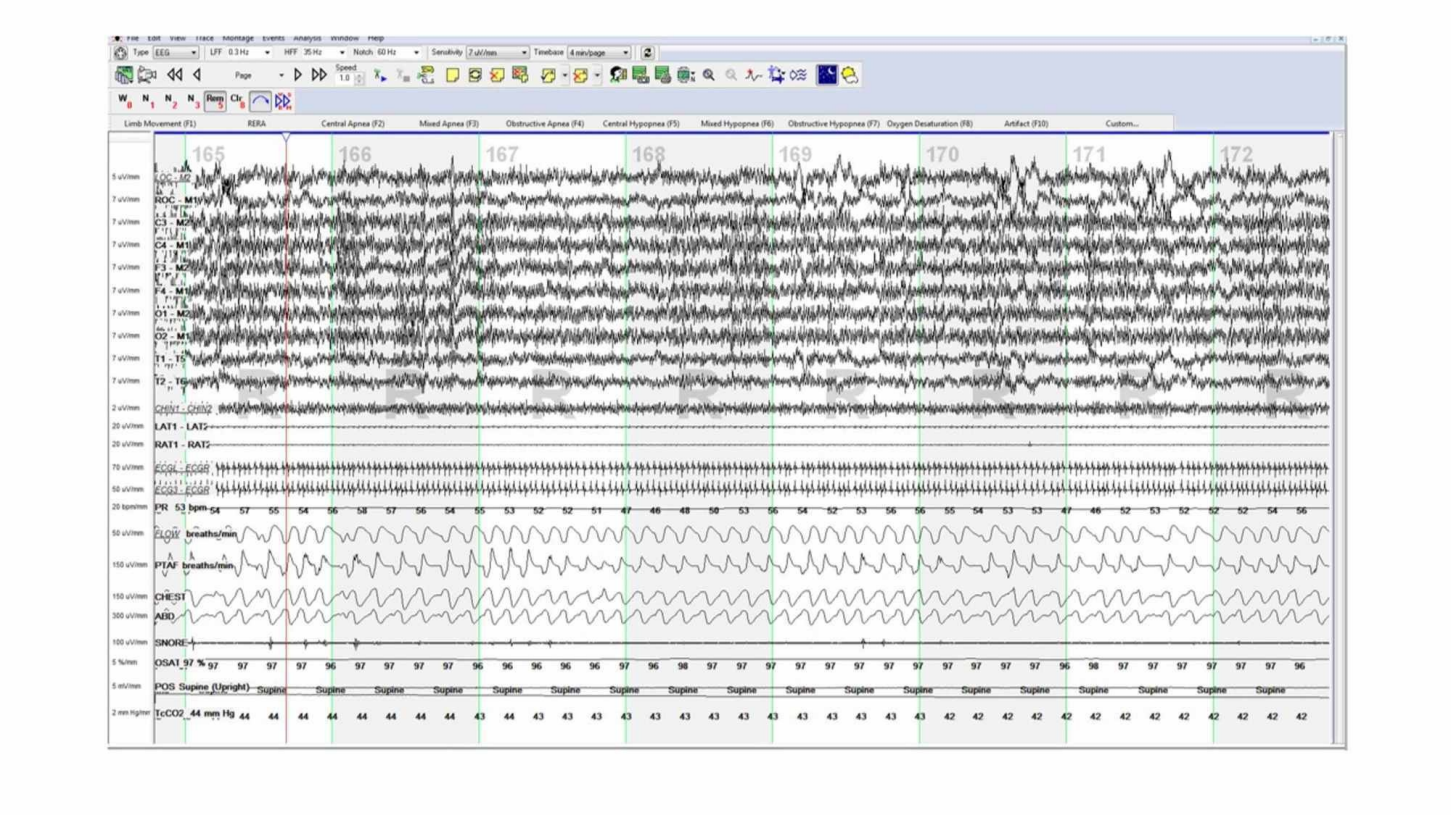
Polysomnogram with the VNS turned OFF did not reveal any obstructive or apneic events. VNS, vagus nerve stimulation.

After discussion with his epileptologist, a decision was made to repeat PSG with the VNS turned off (Figure 2). During the repeat PSG, he had mild intermittent snoring and lowest oxygen saturation was 88%. He had only five obstructive apneas and four central apneas with AHI of 1.2/hour of sleep. At this point, our suspicion that the intermittent VNS activation led to the apneic episodes was confirmed.

## Discussion

In our patient, as it was unclear how much VNS was helping with his seizures, the pros and cons of continuing VNS were discussed with him by his epileptologist and a decision was made to turn the VNS off for a couple of months. He was asked to keep a seizure log with periodic reassessment of his seizure control. If seizures worsened, the VNS pulse generator would be replaced by the newest model which could be turned off at night.

The common side effects of VNS include voice alteration, neck pain, scarring, dysphagia, and cardiac arrhythmia [2]. The effect of VNS on sleep-related breathing was first reported by Malow et al. in 2000 where VNS activation coincided with sleep-related decrease in airflow and effort that did not meet laboratory criteria for hypopneas or apneas. AHI for three of the four subjects was <5/ hour. In one subject with pre-existing OSA, AHI rose from 4 to 11.3. Thus, this study concluded that VNS produced clinically significant adverse effects on sleep-related breathing only in patients with pre-existing OSA [3]. This finding was confirmed again by Marzec et al. in 2003, who studied a sample of 16 patients by PSG three months before and after treatment with VNS [4]. Our case is unique because the patient did not have pre-existing OSA. This suggests that even patients without OSA are prone to developing sleep-related breathing disorders (SBDs).

There are a number of possible mechanisms that can explain the effect of VNS on respiration [3]. These may be central in nature, with an effect on respiratory effort and upper airway patency or more peripheral, with an effect on upper airway musculature. The pathophysiology of central sleep apnea resulting from VNS use is poorly understood, with increased respiratory frequency and decreased amplitude of breaths being associated with VNS. This may be due to VNS-induced tachypnea resulting in diaphragmatic fatigue, with diaphragmatic function taking some time to recover after VNS is stopped, resulting in central apnea [3]. Manipulation of VNS settings such as stimulus intensity, frequency, and cycle times may decrease this respiratory disturbance but the specific adjustment that would be most effective is not known at this time. Few studies have described the presence of vocal cord adduction during the ON period, as evidenced by indirect laryngoscopy, which was associated with the onset of or worsening of a respiratory sleep disorder. Zambrelli et al. evaluated 23 patients with refractory epilepsy and found that 11 patients showed a new mild-to-moderate SBD, and half of all patients with pre-existing OSA showed worsening of SBD after VNS implantation. All the patients with a new-onset OSA showed a pattern of left vocal cord adduction during VNS stimulation. This association was statistically significant, suggesting an important role of laryngeal motility alterations in VNS-induced SBD [5]. Bollu and Sivaraman in 2017 published a case report in which activation of VNS was associated with both obstructive and central apneas in a 47-year-old patient. They described a fairly specific chin EMG artifact as "pearls on a string" which coincided with firing of the VNS [6]. 

## Conclusions

Intermittent VNS can reduce the frequency of seizures in patients with medically refractory epilepsy but can also affect respiration during sleep by a variety of mechanisms. In such a situation, decreasing the VNS output can improve the problem, but may exacerbate seizure activity. Hence, there is a need for clinicians to balance seizure control with VNS-induced sleep disordered breathing. As untreated OSA can worsen seizure frequency, a PSG should be considered after VNS implantation to assess for SBD if there are symptoms of OSA and increased night time arousals. Adjustment of the VNS settings may be required if the onset of respiratory disturbances coincides with the activation of the VNS.
